# Functional heterogeneity of human skin‐resident memory T cells in health and disease

**DOI:** 10.1111/imr.13213

**Published:** 2023-05-05

**Authors:** Johanna Strobl, Muzlifah Haniffa

**Affiliations:** ^1^ Department of Dermatology Medical University of Vienna Vienna Austria; ^2^ CeMM Research Center for Molecular Medicine Vienna Austria; ^3^ Wellcome Sanger Institute Cambridge UK; ^4^ Department of Dermatology and NIHR Newcastle Biomedical Research Centre Newcastle Hospitals NHS Foundation Trust Newcastle upon Tyne UK; ^5^ Biosciences Institute Newcastle University Newcastle upon Tyne UK

**Keywords:** adaptive barrier immunity, allergy, cutaneous T‐cell lymphoma, inflammatory skin diseases, skin cancer, tissue‐resident memory T cells

## Abstract

The human skin is populated by a diverse pool of memory T cells, which can act rapidly in response to pathogens and cancer antigens. Tissue‐resident memory T cells (T_RM_) have been implicated in range of allergic, autoimmune and inflammatory skin diseases. Clonal expansion of cells with T_RM_ properties is also known to contribute to cutaneous T‐cell lymphoma. Here, we review the heterogeneous phenotypes, transcriptional programs, and effector functions of skin T_RM_. We summarize recent studies on T_RM_ formation, longevity, plasticity, and retrograde migration and contextualize the findings to skin T_RM_ and their role in maintaining skin homeostasis and altered functions in skin disease.

## 1 INTRODUCTION

The discovery of tissue‐resident memory T cells (T_RM_) in mice^1^ has initiated a paradigm shift in our understanding of skin immunity: Memory T cells are not merely transiting through skin for surveillance but also form long‐lived sentinels, maintained in the epidermal and dermal compartments and increasingly recognized as central mediators of human cutaneous health and disease.

Cutaneous T_RM_ can mediate local immune protection against invading pathogens and cancer, but have also been implicated in detrimental pathogenic responses after transplantation, in inflammatory diseases and autoimmunity. This review addresses the heterogeneity of T_RM_ development from its early presence in prenatal skin to its continued generation and maintenance in adult healthy skin. While the review is focused on human studies, important murine studies revealing mechanisms of T_RM_ formation and maintenance are discussed as well. Lastly, we highlight the many roles of T_RM_ in a range of allergic, inflammatory and neoplastic skin disorders.

## 2 GENERATION, MAINTENANCE, AND LONGEVITY OF SKIN T_RM_

### 2.1 Prenatal development of skin T cells

Functional populations of tissue‐resident immune cells are seeded in prenatal skin during embryonic life. Some of these myeloid and lymphoid cells become long‐lived and self‐renew in adult murine tissues independently of hematopoietic stem cells.^2^ T‐cell differentiation begins in the developing thymus. Early lymphoid progenitors from human fetal liver and bone marrow migrate into the developing thymus, where they differentiate into mature T cells and acquire a diverse T‐cell receptor (TCR) repertoire.^3^ Studies in mice demonstrated that naive T cells egress from the thymus and seed hematopoietic, lymphoid and nonlymphoid peripheral tissues where further differentiation takes place.^4^ The human embryonic and fetal skin is first populated by innate lymphoid cells (ILCs) and T cells expressing the gamma‐delta T‐cell receptor (TCRγδ). This is followed by αβ‐TCR‐expressing cells from the start of the second trimester at 11–12 post‐conception weeks.^5^, ^6^ Interestingly, humans are born with very few numbers of epidermal T cells,^7^ and T cells isolated from human neonatal foreskin lacked the expression of TRM markers.^8^ This is readily explained by sparsity in foreign antigen load *in utero*.^9^ Despite this, small populations of CLA^+^ T cells are found in fetal skin and one fourth of skin T cells expresses CD45RO (the remainder being largely naive and regulatory T cells).^5^, ^10^ In human fetal skin, αβ‐TCR^+^ T cells predominate with some γδ‐TCR^+^ T cells.^7^ Memory T cells in fetal skin display a TCR of high diversity comparable to adult skin,^3^ but their antigen specificities remain to be studied. In addition, transplacental exposures including microchimerism of maternal cells^11^, ^12^ and prenatal maternal infections have been shown to influence fetal adaptive T‐cell immunity with long‐lasting effects on tissue‐specific immunity and increased risk of inflammatory diseases in later life.^13^

In contrast to other tissues, human fetal skin harbors a small population of hybrid αβ‐γδ T cells, that may contribute to structural skin formation by degradation of extracellular matrix proteins in addition to protective immunity.^14^, ^15^ Although these hybrid cells have been reported to play a role in murine Experimental autoimmune encephalomyelitis by licensing encephalitic Th17 cells,^15^ it remains unclear how these cells may impact human skin inflammatory disorders after their disappearance in post‐natal life. Although the complex process and sequence of skin‐resident T‐cell emergence is beginning to be understood, the consequences of developmental perturbations of these cells on future inflammatory potential are only starting to be explored.

### 2.2 T_RM_ composition and phenotype in adult skin

T_RM_ in human skin emerge primarily after birth following exposure to the external environment and microorganisms. Phenotypically, skin T_RM_ can be discriminated from other CD45RO^+^ memory T‐cell subsets by the lack of lymphatic migratory markers C‐C chemokine receptor type 7 (CCR7) and L‐selectin (CD62L) combined with the expression of surface receptors that promote tissue homing and retention such as cutaneous lymphocyte‐associated antigen (CLA),^16^, ^17^ the sphingosine‐1‐phosphate receptor‐1 (S1PR1)‐antagonist CD69^18^ and the integrin receptors αE (CD103)^1^ and α1β1 (CD49a),^19^ which we detail below.

CLA is expressed by the majority of T cells in healthy human skin and widely used to identify ex‐skin T_RM_ in the circulation.^20^, ^21^ CLA is a carbohydrate modification of P‐selectin glycoprotein ligand‐1 and is a functional ligand for both cell adhesion molecules E‐selectin and P‐selectin^16^ and regulates T‐cell homing to skin. As such, antibody‐targeting of CLA inhibits trans‐endothelial migration of T cells.^22^, ^23^

CD69 is a transmembrane C‐Type lectin rapidly up‐regulated upon TCR stimulation and regarded as an early activation marker for T lymphocytes.^24^ CD69 forms a complex with S1PR1, counteracting S1P‐S1PR1 interaction, which normally attracts T cells from lymphoid organs back to the circulation via a S1P gradient in the bloodstream.^18^, ^25^ CD69 in tissue T cells enables tissue retention and residency. Via the same mechanism of S1PR1‐degradation, CD69 is thought to prevent S1PR1‐induced effector Th1/Th17 differentiation (by JAK2/pSTAT3 activation)^26^ and promote the establishment of T_reg_.^27^

Unlike CD69, CD103 is not essential for the establishment of tissue residency, but rather in long‐term retention of T cells.^28^, ^29^ CD103 forms a heterodimer with integrin β7, mediating binding of the heterodimeric molecule αEβ7 to e‐cadherin on epithelial cells. In turn, integrin β7 may also pair with CD49d to form the heterodimeric receptor α4β7, which is specific to gut homing T cells.^30^ There is little evidence of importance for integrin α4β7 in cutaneous inflammation, due to lack of MAdCAM‐1 receptor expression in skin and low rates of cutaneous adverse events following treatment with α4β7 (vedolizumab).^31^

Early on, CD103 and αEβ7 had been used as a specific marker for intestinal intraepithelial T cells but was soon found to be expressed by other cell types, including dendritic cells, mast cells, and ILCs.^32^ Although up‐regulation of αEβ7 was shown to promote retention of T_RM_ in epithelial tissues,^33^ the presence of CD103^+^ T cells in organs with low e‐cadherin expression argues for more complex consequences on T‐cell homing that are yet to be defined. In addition, whether αEβ7 may possess direct cytotoxic functions to lyse e‐cadherin expressing cells, including keratinocytes is unclear.^34^, ^35^

Similarly, integrin CD49a expression defines T_RM_ with cytotoxic functions and the ability to produce interferon‐gamma (IFN‐γ), perforin and granzyme B upon appropriate stimulation.^19^ CD49a is the α‐subunit of the α1β1 integrin receptor, which binds to collagen IV of the basement membrane, resulting in accumulation of CD49a^+^ T_RM_ in the epidermis, where they are well positioned to fight viral pathogens.^36^ CD49a is expressed by the majority of murine virus‐specific T_RM_, but only 14% of human skin T cells, arguing for a distinct subset of cytotoxic T_RM_ with defined function within the heterogeneous skin T_RM_ population.^37^

Additional skin homing receptors reported to mark skin T_RM_ include the C‐C chemokine receptor (CCR) types including CCR4, CCR5, CCR8 and CCR10, as well as CXCR3.^38^, ^39^, ^40^, ^41^, ^42^, ^43^ CCR4 is the receptor for several chemokines expressed by antigen presenting cells, including CCL2, CCL17, and CCL22.^44^ It is a skin‐specific T‐cell homing molecule, expressed highly on cutaneous T_reg_, Th2 and Th17 T_RM_,^21^, ^45^ but there exist also a population of CLA^hi^ CCR4^+^ central memory T cells in the bloodstream.^38^, ^46^ A CCR4‐antibody mediating antibody‐dependent cellular cytotoxicity is used for treatment of aggressive cutaneous T‐cell lymphoma (CTCL).^47^

CCR5, widely known as an entry receptor for HIV‐1,^48^ has been described as a an epitope marking skin resident T_reg_.^39^, ^49^ It remains to be determined whether HIV entry into skin resident T_reg_ is increased during skin inflammation^50^ among people living with HIV. The receptor CCR8 promotes skin homing by binding of CCL1, a chemokine expressed by Langerhans cells and dermal microvessels.^51^ CCR8 is a marker of both epidermal and dermal T_RM_ and is preferentially expressed on CD4^+^ T_RM_.^40^ CCR8^+^ and CRR8^−^ skin T‐cell populations were found to be clonally unrelated. CCR8^−^ T cells showed cytotoxic effector functions and express T‐bet but CCR8^+^ T cells are long‐lived T_RM_ with a diverse cytokine repertoire.^40^ CCR10 is another essential skin T‐cell homing molecule and binds to its ligands CCL27 and CCL28, produced by epithelial cells.^41^, ^52^ CCR10 is also expressed by melanocytes and CCR10‐transduced melanoma cell lines possess superior metastatic potential.^53^ In addition, entry of T_RM_ into the epidermis involves the interaction of CXCR3 on T cells with its ligands CXCL9 and CXCL10.^54^, ^55^, ^56^ CXCR3 is preferentially expressed by CD8^+^ T_RM_ and IFN‐γ‐producing CD4^+^ T_RM_.^57^, ^58^

**FIGURE 1:**
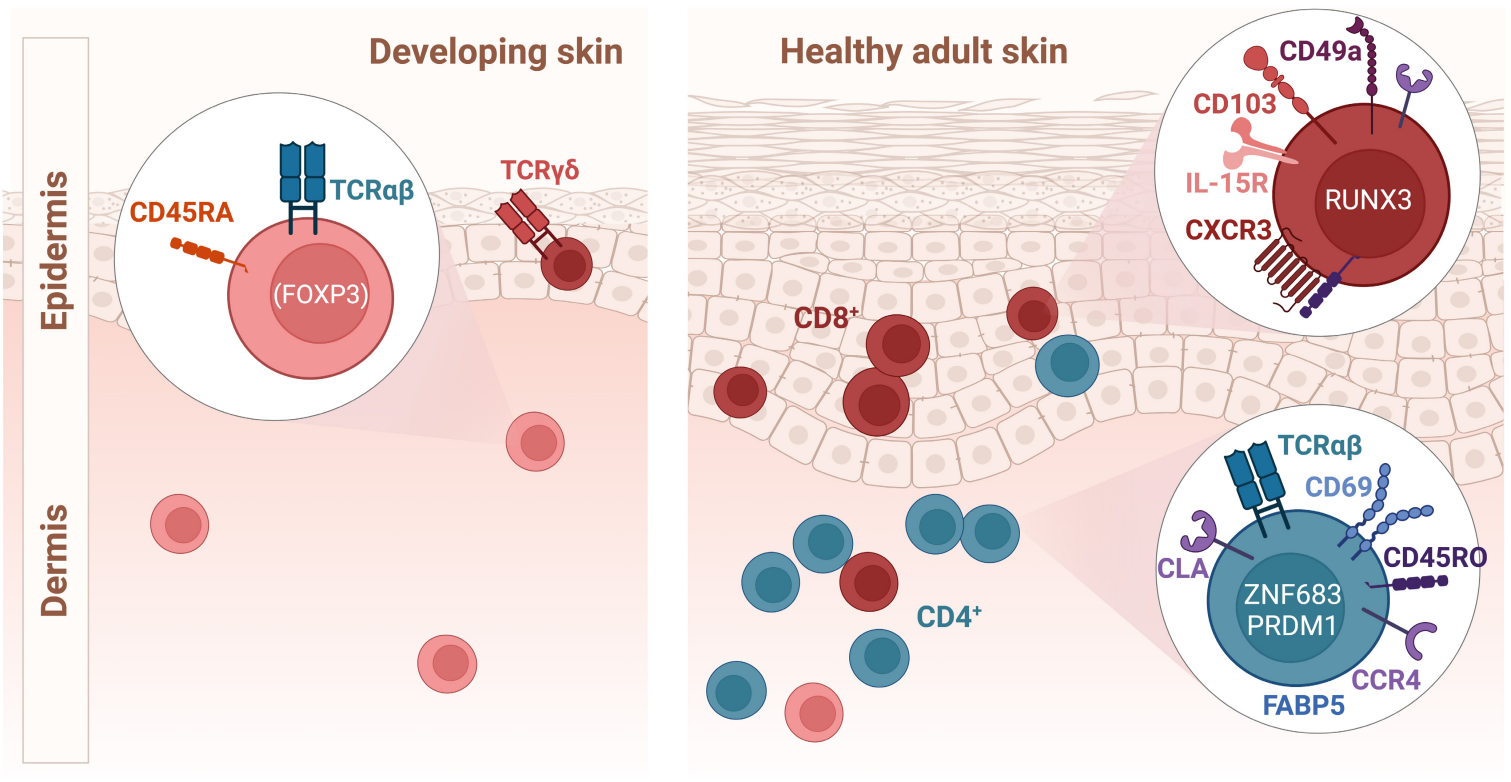
Heterogeneity of T_RM_ in developing and adult human skin. Schematic showing commonly expressed surface receptors and transcription factors of dermal and epidermal T‐cell populations in developing human skin and healthy adult skin.

This above‐described set of surface molecules is expressed by T_RM_ in the two skin compartments, the epidermis and dermis, to varying degrees (Figure 1). Unlike in mice, where epidermal CD8^+^CD103^+^T_RM_ predominate, the majority of T_RM_ in healthy adult human skin is localized to the dermis, are CD4‐positive, and co‐expresses CD69. The CD4/CD8 skin T_RM_ ratio has been found to decrease with age independent of ethnicity and geographical background.^59^ Human epidermis is mainly populated by CD8^+^ T_RM_, which, in contrast to murine dendritic epidermal T cells (DETC), do not extend dendritic processes. The integrin CD103 is expressed by a fraction of human dermal T_RM_ and >60% of epidermal T_RM_.^8^

Besides epidermal and dermal T cells, lymphocytes in subcutaneous and dermal white adipose tissue have received some attention in recent years, mainly due to their potential role in assisting cutaneous pathogen clearance.^60^ However, the function of T_RM_ in subcutaneous and dermal white adipose tissues is mainly considered to be regulatory, due to the high density of T_regs_ and innate immune cells in these tissues.^61^ Nevertheless, the subcutaneous adipose tissue also harbors a large number of memory T cells of which 25% are CD69^+^CD103^+/−^,^62^ representing a significant pool of T_RM_ in the layer directly underneath the skin. Their potential influence on adaptive cutaneous immunity and skin inflammation remains to be investigated.

### 2.3 The T_RM_ ‐specific transcriptional program is initiated in response to antigen

In response to cutaneous antigen encounter, T_RM_ may develop both via circulating memory T cells recruited from blood as well as arise from pre‐existing T_RM_ populations within the skin. Interestingly, T_RM_ retain some level of plasticity as demonstrated in mice by their potential to trans‐differentiate into effector memory (T_EM_) and central memory T cells (T_CM_).^63^ Distinct functional phenotypes of human T_RM_ can be derived based on the type of circulating T cells recruited into skin. Central memory T cells are highly efficient precursors of T_RM_ and generate the largest number of FOXP3^+^ T_RM_.^64^ Migratory memory T cell‐derived T_RM_ preferentially express IL‐17A and effector memory T cells display higher levels of CXCR3 and give rise to IFN‐γ^+^ T_RM_. Skin infection results in the generation of (i) short‐lived KLRG1^+^ terminal effector cells and (ii) KLRG1^−^IL‐7Rα^hi^ memory precursor effector cells in murine lymphoid tissues.^65^, ^66^ While terminal effector T cells in mouse skin rapidly undergo apoptosis after pathogen clearance, memory effector T cells loose KLGR1 expression and differentiate to long‐lived skin T_RM_.^54^, ^67^, ^68^ This process of murine T_RM_ formation seems to be dependent on the transcription factor aryl hydrocarbon receptor (AhR), which is also highly expressed on human skin and gastrointestinal T_RM_, and is a target for the topical treatment of psoriasis.^69^, ^70^, ^71^ In both mouse and human tissues, local signaling by IL‐7, IL‐15^72^ or transforming growth factor‐beta (TGF‐β)^73^, ^74^ is required for the instruction of a tightly regulated transcriptional program resulting in T_RM_ establishment.^75^ Responsiveness of T cells to local TGF‐β is preceded by downregulation of EOMES and TBET.^76^ Transcription factors of murine CD8^+^ T_RM_ are well‐studied, with HOBIT (ZNF683) and BLIMP‐1 (encoded by PRDM1) defining early commitment to a resident phenotype.^77^ The human homologue to HOBIT is not found in T_RM_ of all organs.^78^ Binding of HOBIT or BLIMP‐1 to TCF1 and KLF2 prevents expression of downstream genes for CCR7 and CD62L, instructing tissue residency.^78^, ^79^ HOBIT and BLIMP‐1 have also been implicated in the transcriptional program of pro‐inflammatory CD4^+^ T cells in human cytomegalovirus (CMV) infection and gastrointestinal inflammation.^80^, ^81^ High proportions of CD4^+^ T_RM_ expressing these transcription factors in skin pathology seem likely, but have not formally been investigated.

T_RM_ subset differentiation is also guided by cytokines in the local tissue microenvironment. For example, IL‐23 signaling by myeloid cells results in formation of Th17 cells in human and murine skin,^82^ likely by the induction of BLIMP‐1.^83^ Exposure to TGF‐β increases the expression of αEβ7 by CD8+ T cells which co‐express RUNX3, a marker for long‐term skin T_RM_.^33^, ^84^, ^85^ Importantly, generation of new T_RM_ increases the overall tissue T‐cell memory pool without displacement of pre‐existing populations. In line with this, maintenance of diversity within the cutaneous T‐cell pool increases with age and at a higher level to peripheral blood memory T‐cell pool.^59^ Overall, the generation of T_RM_ in several waves establishes a durable, decentralized and diverse defense system at the skin barrier site.

### 2.4 Maintenance and re‐challenge of skin T_RM_

The phenotypic and transcriptional distinction of T_RM_ by tissue type^86^, ^87^ suggests that local environmental clues are responsible for their regional adaptation. For skin, which is a lipid‐rich environment, metabolites derived from keratinocytes,^88^ signaling via the AhR and fatty acid uptake via fatty acid binding proteins (FABP) followed by fatty acid oxidation^89^ contribute to CD8^+^ T_RM_ survival and longevity. This metabolic feature seems to be partially regulated by PPAR‐gamma^90^ and specific to skin T_RM_,^91^ suggesting the epidermal FABP (FABP5) as a skin‐specific surface marker for long‐lived T_RM_. Besides meeting metabolic requirements, the skin environment contains a plethora of locally residing antigen presenting cells producing chemokines and cytokines including IL‐7, IL‐15, and TGF‐β for T_RM_ maintenance.^92^, ^93^ Cross talk of T_RM_ with antigen presenting cells and stromal cells is extensively reviewed elsewhere.^94^

In steady state, the percentage of skin T_RM_ proliferating within a week, detected as bromodeoxyuridine (BrDU)‐uptaking cells, is below 5%.^1^ This number is lower than that of T_RM_ in other tissues, for example the brain parenchyma, which suggests superior survival capacity of individual skin T_RM_ in a resting state due to adaptation to local nutrient availability.^95^ Upon re‐challenge with viral antigens such as Herpes Simplex Virus or Vaccinia Virus (VV), murine epidermal T_RM_ divide and expand locally, giving rise to new CD103^+^ T cells, but not to CD103^−^ T cells.^96^ Intriguingly, proliferation occurs exclusively at the site of re‐challenge and is specific to its cognate antigen. The extreme specialization to the epidermal niche and site‐specific pathogens provides an efficient, targeted memory response without any need for systemic involvement.

### 2.5 Longevity of skin T_RM_

To provide life‐long protection against previously encountered pathogens, the diverse T_RM_ population is maintained by a unique metabolic program supported by the local environment within the skin. Whereas naive T cells are dependent on TCR stimulation for survival, longevity of LCMV‐induced CD8^+^ T_RM_ did not require viral antigen persistence nor TCR‐signaling.^97^ Studies on the skin of individuals after allogeneic hematopoietic stem cell transplantation (HSCT) suggest that T_RM_ survive in human skin for decades without replenishment from the circulatory pool. Recipient‐derived T_RM_ form surprisingly large populations in the skin of patients after HSCT, despite myeloablative treatment and full donor chimerism of peripheral blood T cells.^84^, ^98^ Similarly, intravenous treatment with the anti‐CD52‐antibody alemtuzumab, which depletes all lymphocytes from peripheral blood, left intact a diverse population of skin T_RM_ with effector functions.^99^ Molecular factors implicated in the long‐term survival of T_RM_ in skin include constitutive STAT5‐pathway activation and Wnt/TCF‐1 signaling leading to the expression of anti‐apoptotic molecules such as Bcl‐2.^100^, ^101^, ^102^ In line with this, Bcl‐2 overexpression was also found in pro‐inflammatory skin T cells in steroid‐refractory skin inflammation.^103^

### 2.6 Migration of skin T_RM_

Unlike previously thought, skin T_RM_ do not remain completely non‐migratory during their life cycle.^63^ Indeed, in murine parabiosis experiments, 15%–30% of circulating memory T cells were determined ex‐T_RM_.^97^ It is suggested that CD4^+^ skin T cells have a superior capacity to re‐enter the circulation, compared to their CD8^+^ counterparts.^104^ Studies in humanized mice and healthy human volunteers have revealed a small but stable population of ex‐T_RM_ populating the circulatory T‐cell pool and accounting for roughly 1% of CD4^+^ blood T cells.^20^, ^105^ Re‐circulating former skin T_RM_ down‐regulate CD69 to exit the tissue and are characterized by the expression of CLA and CD103. Ex‐T_RM_ retain their predilection to tissue of origin and largely keep the transcriptional signature of their sessile tissue resident counterparts, for example the expression of key transcription factor GATA3 and cytoplasmatic FABP5 by ex‐T_RM_ from skin.^106^ In heathy individuals, the circulating CLA^+^ T‐cell population is thought to remain stable via continuous outflow of ex‐T_RM_ from the skin compartment.^105^, ^107^ However, this population expands in the peripheral blood during graft‐versus‐host skin inflammation and viral skin infections.^105^ Importantly, it is likely that ex‐T_RM_ seed distant organs, distinct from their tissue of origin, where they fulfill functions that are not served by seeding of naive or circulating memory T‐cell subsets.^108^ A study in humans and rhesus macaques has identified distinct clonotypes and functional properties of intravascular CD8^+^ T cells. These cells present in thoracic duct lymph can be distinguished by distinct epigenetic potential and the expression of skin‐residency markers CD103, CCR5, CXCR3, CXCR5. CD103^+^CD27^−^ “ex T_RM_” T cells drained from nonlymphoid tissues had progenitor‐like properties and rarely expressed cytolytic molecules.^109^

Together, circulating T_RM_ may explain recall responses for several previously unexplained immune phenomena including spread of skin inflammation to other skin sites (Koebner phenomenon), involvement of joints and distant tissues in psoriasis, and skin inflammation relating to systemic disease such as pyoderma gangrenosum associated with inflammatory bowel disease. In addition, as CLA^+^ T‐cell population in blood reflect inflammatory processes occurring in skin, these cells can be used for diagnostics and therapeutic targets in dermatologic conditions.

## 3 FUNCTIONS OF SKIN T_RM_

### 3.1 Barrier defense, wound healing, and protection against infection

The skin is one of the largest barrier organs in the human body. Due to its dense population with T_RM_, it is no coincidence that the pivotal role of T_RM_ in barrier immunity was initially identified in the skin of mice challenged with viral antigens.^1^, ^110^, ^111^ Not only are skin T_RM_ much more abundant than circulating T cells in the human body,^8^ their ability to mediate pathogen clearance at local sites of infection is superior to that of their circulating memory counterparts.^112^ Therefore, T_RM_ are starting to steal the spotlight from circulating memory T cells in vaccine design and other strategies to enhance immunity to pathogens.^87^

The central role of T_RM_ in barrier immunity to infections was demonstrated in viral, bacterial, fungal, and parasitic infections: CD8^+^ effector T cells are rapidly recruited to murine skin following local challenge with VV, leading to protective immunity of the entire skin surface.^113^ CD8^+^ T_RM_ generated from effector memory populations provide initial containment of pathogens in re‐infection with herpes viruses.^1^, ^114^, ^115^ Dengue virus infection results in generation of both CD4^+^ and CD8^+^ dengue‐specific T_RM_.^116^ In *C. albicans* skin infection, CD4^+^ T_RM_ produce IL‐17 to rapidly clear recurrent infections.^117^ *Leishmania*‐specific CD4^+^ T_RM_ produce IFN‐γ resulting in protection against reinfection with *L. major*.^118^ Interestingly, in some vector‐borne skin infection T_RM_ function may be impaired, as haemophagous pathogen vectors have adapted to prolong feeding by suppressing local immune reactions. This results in increased transmission of *Borrelia burgdorferi* in transmission by tick vectors.^119^ Similarly, in *Aedes aegypti* bites, mosquito saliva suppressed pro‐inflammatory responses, resulting in regulatory T cells and Th2 polarization in the skin.^120^

In clearance of infection and in wound healing, tissue‐resident T_regs_ come into action: Directly following barrier breach, skin‐resident T_regs_ initially promote inflammation at the keratinocyte layer.^121^ In burn injury, skin T‐cell populations shift from a resident phenotype to a circulating homing marker profile.^122^ In later stages of epithelial injury, skin‐resident γδ‐T cells and BATF^+^CCR8^+^ skin T_regs_ are thought to promote physiological wound healing by partaking in a tightly regulated response comprising pro‐/anti‐inflammatory signals and growth factors,^123^, ^124^ whereas increased numbers of TNF‐α‐producing CD8^+^ memory T cells were found in hypertrophic (keloid) scars.^125^

As first line of defense, T_RM_ are considered important targets in vaccine design,^126^ and the route of vaccine administration will be critical to promote the generation of protective CD8^+^ T_RM_ in barrier tissues. In *orthopoxvirus* and *modified vaccinia Ankara virus* vaccination, administration via skin scarification resulted in superior skin T_RM_‐mediated immunity compared with classical vaccination routes, including subcutaneous or intramuscular vaccination.^127^, ^128^ This has implications for vaccinations against mucosal and respiratory infections, including COVID‐19, where alternative vaccine administration routes for better protective immunity are being evaluated.^129^, ^130^

Although attracting some attention in recent years, sexual dimorphism in human infectious diseases is poorly understood and severely understudied in skin. After mild COVID‐19 infection, blood T cells of male recoverees displayed higher activation of CD8^+^ memory subsets and higher IL‐15 responses upon influenza vaccination.^131^ This likely results in stronger cues for T_RM_ differentiation providing a first indication that efficiency of infection‐generated memory T cells may be dependent on sex.

Overall, the emergence of COVID‐19 as a pandemic virus has rapidly increased global knowledge of T_RM_ in respiratory infection,^132^, ^133^ including studies into long‐term systemic T‐cell aberrations^134^ and detailed mechanistic understanding of vaccination response at mucosal sites.^135^ We anticipate many of the discoveries on skin T_RM_ to be relevant for T_RM_ in other barrier tissues supporting the need and importance of skin research.

### 3.2 Anti‐tumor immunity

The diverse T_RM_ pool protecting the skin barrier can only partially recover once depleted. This has been demonstrated in people living with HIV after late start of antiretroviral therapy, where despite recovery of circulatory CD4^+^ T‐cell numbers, skin T_RM_ failed to resurge.^136^ As a consequence, irreversible loss of CXCR3^+^ T_RM_ increased the risk of HPV‐related cancer in these individuals.

The role of peri‐tumoral T cells in anti‐tumor immunity has been demonstrated for a large number of cancers.^137^ In skin cancers and other solid tumors of barrier tissues, T_RM_ are present as CD8^+^CD103^+^CD49a^+^ tumor‐infiltrating lymphocytes with effector (memory) functions^138^ and are generally associated with good outcomes.^139^, ^140^ Studies in transplant recipients suggest that tumor‐specific T_RM_ are recruited to tissues upon occurrence of malignant cells where they form long‐lived populations.^141^ Tumor‐specific T_RM_ are present in lymph nodes^142^ and constantly surveil the skin in what has been termed the cancer‐immune‐equilibrium.^143^, ^144^ Antitumoral functions include direct tumor cell lysis by cytotoxic molecules^145^ and stimulation of de novo cytotoxic T‐cell generation in lymph nodes via dendritic cell antigen spreading.^146^ Fitting with anti‐tumor efficacy of oncolytic virus therapy,^147^ natural virus‐specific CD8^+^ T_RM_ were found in peri‐tumoral tissues.^148^ More recently, also CD4^+^ virus‐specific T_RM_ were identified in lung and colorectal cancer.^149^ As skin cancers often associate with localized dysbiosis or viral infection, antigen specificity of peri‐tumoral T_RM_ and their role in cancer immunotherapy will need to be addressed by future studies.

The presence of melanoma‐specific T_RM_ conveys protective immunity to melanocytic skin cancer.^150^ Nonmalignant melanocytic lesions contain increased numbers of CD103^+^CD8^+^ T_RM_. In the melanoma tumor microenvironment, abundant CD69^+^ T cells are found.^151^ Importantly, CD69^+^CD103^+^ tumor‐resident CD8^+^ T cells and local IL‐15 production were associated with response to immune checkpoint inhibitors.^139^ CD8^+^ T_RM_ exhibit high levels of PD‐1, CTLA‐4, and LAG‐3, and a recent review is dedicated to their crucial role in melanoma immunotherapy.^152^ Conversely, CD69^+^CD103^+^ T_RM_ exhibited increased IL‐10 production in cutaneous squamous cell carcinoma, a common non‐melanoma skin cancer (NMSC), probably representing an exhausted dysfunctional T‐cell subset.^153^, ^154^ However, some ability for IFN‐γ and TNF‐α cytokine production was retained and high expression of immune checkpoints could be observed, suggesting a complex role for T_RM_ in NMSC and its treatment.

Increased prevalence of NMSC is observed in organ transplant recipients,^155^ due to decreased cytotoxic T_RM_ by long‐term immunosuppressive therapy.^156^ Interestingly, immunosuppressive treatment of inflammatory skin diseases including psoriasis does not seem to increase risk of melanoma or NMSC.^157^ However, the difference in potency and dose of immunosuppressive drugs used in solid organ transplants and in psoriasis and the absence of large observational studies on psoriasis patients receiving immunosuppression may contribute to the apparent difference in susceptibility to NMSC. One can only speculate whether T_RM_ in the skin of patients with inflammatory skin diseases also provide superior local protection against skin cancer.

## 4 T_RM_ IN DISEASE PATHOGENESIS

### 4.1 Inflammatory skin diseases

#### 4.1.1 Drug reactions and contact allergic disease

One of the first inflammatory conditions thought to be mediated by local resident T cells (Table 1) was fixed drug eruption (FDE), a localized form of drug hypersensitivity characterized by recurrent inflammation at the exact same skin site upon systemic exposure to the causative agent.^158^ In FDE, intradermal CD103 and CD49a‐expressing CD8^+^ effector memory T cells show cytotoxicity and increased production of IFN‐γ.^159^ In addition to high numbers of T_RM_ in healed FDE, active FDE lesions also contain high percentages of T cells with a central memory phenotype (T_CM_). Interestingly, TCR sequence overlap suggests that this population is not recruited from circulatory T_CM_ but rather trans‐differentiates from local cytotoxic T_RM_.^160^

**TABLE 1:** Overview of major T cell‐associated inflammatory skin diseases.

Disease	T‐cell phenotype	Cytokine responses	Location of T‐cell infiltrate
Fixed drug eruption	Drug‐specific CD49a^+^CD103^+^CD8^+^ T_RM_ and T_CM_	Th1; cytotoxic	Dermis
Allergic contact dermatitis	CD8^+^T_RM_ CCR10^+^CD4^+^T_RM_	Th2	Dermis
Delayed‐type hypersensitivity (SJS, TEN)	Drug‐specific clonally expanded CD8^+^T_RM_	Granzyme B	Epidermis
Atopic dermatitis	CCR8^+^CRTH2^+^CD4^+^ T cells/T_RM_	Th2A; Th22; Th9	Dermis
Psoriasis	CCR6^+^CD103^+^CD49a^−^CD8^+^T_RM_; CD4^+^T_RM_	IL‐17A; IL‐22	Epidermis
Polymorphic light eruption	CD103^+^CD49a^+^CD8^+^T_RM_	Cytotoxic	Dermis
Graft‐versus‐host disease	Host CD103^+^RUNX3^+^CD8^+^T_RM_; donor CD8^+^ T cells	Th1; Th2; cytotoxic	Dermis
Vitiligo	Melanocyte‐specific CXCR3^+^CD49a^+^CD8^+^ T_RM_	Cytotoxic	Epidermis
Alopecia areata	CD103^+^CD8^+^T_RM_	Cytotoxic; Th1	Hair follicle
Cutaneous lupus erythematosus	CD4^+^T_RM_	Th1; Th2	Dermis
Dermatomyositis	CCR10^+^CD4^+^T_RM_; CCR7^+^T_CM_; CCR4^+^CD4^+^T_RM_ (muscle)	Th17; IFN‐b; Th2 (muscle)	Dermis; muscle tissue
Systemic sclerosis	Th2, CD8^+^T_RM_	IL‐13	Dermis/subcutis

Skin T_RM_ are also implicated in the pathogenesis of other drug‐induced or contact allergic dermatoses. Stevens–Johnson syndrome and toxic epidermal necrolysis (TEN) are severe delayed‐type drug hypersensitivity reactions resulting in blister formation and high mortality. In both FDE and blistering hypersensitivity conditions, drug‐specific clonally expanded T cells mediate keratinocyte apoptosis by perforin and granzyme release from cytotoxic T_RM_.^161^, ^162^, ^163^ While the accumulation of skin T_RM_ mediating disease pathology is well described, it remains enigmatic how drug‐specific T_RM_ form at a localized skin site in FDE, or widely distribute across the body surface prior to reactivation in TEN. The presence of specific HLA alleles followed by a “second hit” with viral skin infections is discussed as a potential mechanism leading to generalized drug hypersensitivity conditions.^164^, ^165^ Although this ‘two‐hit’ mechanism has also been demonstrated for amoxicillin‐induced maculopapular rash in association with EBV infection,^166^ the T‐cell infiltrate comprise CD4^+^ Th1 and Th2 effector memory T cells and smaller percentages of cytotoxic T cells in contrast to T_RM_ in TEN lesions.^162^, ^167^ This is in line with a different clinical and histological presentation without any blister formation and dermal rather than epidermal lymphocytic infiltration.^167^ In allergic contact dermatitis (ACD), active lesions contain a mixed CD4^+^/CD8^+^ lymphocytic infiltrate with persisting T cells preferentially expressing CD4 and CCR10.^168^ Long‐term immunological memory in murine hapten‐induced ACD was shown to be mediated by CD4^+^ T_RM_ and initially confined to sensitized body areas until re‐challenge.^169^ Recently however, studies in mice found the magnitude of flares depending on numbers of epidermal CD8^+^ T_RM_ and increasing upon elimination of CD4^+^ T_RM_.^170^, ^171^ Additional translational studies will be required to dissect differences of ACD mouse models and human contact dermatitis.

#### 4.1.2 Psoriasis and atopic dermatitis

T_RM_ have been reported in lesional skin of the two common inflammatory skin disorders, psoriasis, and atopic dermatitis (AD). Local immune memory in psoriasis is well understood: CD8^+^ CD103^+^CD49a‐T_RM_ express CCR6 and produce IL‐17A, and CD4^+^ T_RM_ produce IL‐22. Both are primarily αβTCR‐expressing cell communities, which are retained in the epidermal compartment of healed psoriasis lesions.^19^, ^172^, ^173^ A recent study suggests that loss of regulatory signals via the CCL27/CCR10‐axis augments pro‐inflammatory signaling by T_RM_.^174^ Interestingly, these cells are even present in nonlesional skin of patients with psoriasis.^175^ Psoriasis may also prominently affect the joints in psoriasis arthritis. Here, CXCL9/10 are over‐expressed in synovial fluid of affected patients and CXCR3 is expressed by clonally expanded synovial CD8^+^ T_RM_ producing IL‐17A.^176^, ^177^ Notably, psoriasis arthritis may be distinguished from skin‐confined psoriasis by the presence of CLA^+^CCR4^+^CCR10^+^ T cells in peripheral blood, suggesting migration of pathogenic T‐cell clones across tissue compartments.^106^ These cells retained a tissue resident transcriptional profile and type‐17 cytokine production.^106^

In AD, signaling by keratinocytes induces CCR8‐mediated homing of T cells expressing CRTH2, a marker also described on T cells in allergic asthma.^178^, ^179^ Th2, specifically Th2A cells,^180^ and Th22 cells dominate the inflammatory infiltrate in AD skin.^181^ Other T helper cell subsets have been described specifically in AD, including Th9 cells.^182^ AD lesions harbor a highly polyclonal TCR repertoire, which are also shared with peripheral blood and nonlesional skin. Persistence of the prevalent TCR clones months after therapy suggests generation of long‐lived T_RM_ in AD propagation.^183^ Circulating Th2‐ and Th‐22‐polarized CLA^+^ T cells, most likely ex‐T_RM_, has been observed in adult AD as potential disease biomarkers.^184^, ^185^ Recently, CLA^+^ T cells were identified as superior producers of Th2 cytokines in the blood of a murine AD model.^186^ Local microbiome disruption and *Staphylococcus aureus* colonization in AD have been shown to induce epithelial cytokine production, including IL‐33 and thymic stromal lymphopoietin which can modulate skin T_RM_ phenotype and function.^187^, ^188^

#### 4.1.3 Polymorphic light eruption and graft‐versus‐host disease

Other skin disorders reported to be mediated by skin T_RM_ include chronic recurring forms of allergic disease are polymorphic light eruptions, which occur seasonally at sites of UV‐exposure and cutaneous graft‐versus‐host disease (cGVHD) following allogeneic HSCT. The majority of cells in polymorphic light eruptions are cytotoxic CD8^+^ T_RM_, characterized by the expression of CD69, CD103, and CD49a, which are induced by macrophage‐derived IL‐15.^189^ In cGVHD, proliferation of conditioning treatment‐resistant host T_RM_ within epithelial tissues^84^, ^98^ and the subsequent influx of circulating donor‐derived T cells, which rapidly acquire a T_RM_ phenotype, creates an interesting immunological situation of “new” and “old” T_RM_ populations of different genetic background contributing to skin inflammation.^190^ In both polymorphic light eruption and cGVHD, a shift in local microbial communities following sunlight exposure^191^ and conditioning regime treatment,^192^ respectively, resulting in epithelial barrier disruption and cytokine release, have been implicated in disease pathogenesis.

#### 4.1.4 Autoimmune skin disorders

Skin T_RM_ have been reported to mediate autoimmune disorders such as alopecia areata (AA), a common autoimmune condition resulting in non‐scarring hair loss, cutaneous lupus erythematosus (CLE), dermatomyositis, and vitiligo. For fibrotic autoimmune skin conditions, the pathophysiological role of T_RM_ remains incompletely understood, but evidence from murine models suggests roles for IL31‐ and fibroblast‐mediated Th2‐polarization in systemic sclerosis, and IL‐13‐producing CD8^+^T_RM_ were found in skin samples of affected patients.^193^, ^194^, ^195^

In AA, the population of circulating CLA^+^ IL‐13 and IL‐22‐producing T cells is expanded and correlates with disease severity.^196^, ^197^ In a mouse model of AA, cytotoxic CD8^+^NKG2D^+^ T cells and local IFN‐γ production led to loss of hair follicle immune privilege and pathology was prevented by antibody‐mediated blockade of IL‐15 receptor and CXCR3.^42^, ^198^ Hair follicle‐derived autoantigens are suspected to drive antigen‐specific cytotoxic T‐cell responses against the follicle's matrix epithelium.^199^ In line with this, clonally expanded CD8^+^ and CD4^+^ T_RM_ expressing CD69 and CD103 were found in human AA lesions.^200^, ^201^ Interestingly, spatial localization of T_RM_ within the hair follicle determined clinical pathology in AA in contrast to scarring forms of alopecia. As TCR specificities and migration marker profiles of alopecia‐associated T_RM_ are currently unknown, open questions remain how T_RM_s are instructed to migrate to specific hair follicle regions.

CLE comprises a diverse group of localized and disseminated autoimmune skin eruptions, which may also be associated with scarring and non‐scarring hair loss.^202^, ^203^ Skin lesions can be classified as acute, subacute, and chronic CLE, with further subvariants. Although autoantibodies, B lymphocytes, and innate immune cell types are more central to pathogenesis of CLE, T cells are considered important in disease propagation.^204^ Persistence of chronic CLE subtypes with weak response to B cell depletion may be strongly driven by the presence of clonal CD4^+^ T_RM_ populations.^205^, ^206^ In addition to anti‐nucleosome antibodies, circulating TCR clones in systemic lupus erythematosus and lupus nephritis were shown to be specific against nucleosomal peptides of histone regions, while specificities in CLE remain unknown.^207^, ^208^ Recent work in a murine CLE model suggests that initial skin infiltration with Th2 cells and re‐polarization to Th1 T_RM_ is required for persistence of CLE skin eruptions. Th1‐polarized T_RM_ produce IFN‐γ and maintain their phenotype in disease recurrence.^204^, ^209^ As in addition IL‐15‐induced circulating CD4^+^ T cells with cytotoxic properties were recently found in systemic lupus erythematosus,^210^ future studies of skin T cells in CLE may offer interesting insights into epigenetic potential of CD4^+^ T_RM_.

Dermatomyositis is a systemic autoimmune disorder affecting multiple organ systems. Divergent populations of T helper cells were found in skin and muscle tissues of affected patients: Muscle‐infiltrating T cells were largely CCR4^+^ Th2 T_RM_ producing IL‐4, whereas skin harbored CCR10^+^ Th17 cells.^211^ Surprisingly, a recent study detected phenotypic central memory T cells (CCR7^+^) producing type‐1 interferons (IFN‐β) as major T‐cell type in dermatomyositis skin lesions,^212^ which stands in contrast to the T_RM_ phenotype found in muscle and in previous skin studies. Furthermore, circulating CD69^+^ T cells correlated with dermatomyositis disease severity. It remains to be investigated whether those represent T cells recently emigrated from affected tissues or are homing to skin and muscle from peripheral blood.

Vitiligo is a depigmenting inflammatory skin disease considered to be caused by melanocyte‐specific cytotoxic CXCR3^+^CD49a^+^CD8^+^ T_RM_.^19^, ^58^ Unlike previously thought, the perilesional immune milieu at the border of active vitiligo seems to be quite complex, with Th1‐ and Th2‐related cytokine secretion increasing pro‐inflammatory signaling by keratinocytes and melanocytes, including the release of IL‐15.^213^, ^214^ This promotes activation of NKG2D^+^ CD8^+^ effector memory T cells releasing IFN‐γ and TNF‐α.^215^ Therapeutically, inhibition of T_RM_ formation by targeting the IL15R subunit CD122 reverses disease pathology and induces skin‐repigmentation.^216^ Local and systemic JAK1/2 inhibition does not deplete T_RM_ but may strongly inhibit epidermal activation.^213^, ^217^ Work performed in a mouse model suggests that in non‐progressive vitiligo, disease is maintained both by local T_RM_ and circulating T_CM_.^218^ This contrast to other T_RM_‐mediated diseases where disease is maintained locally, including psoriasis.

It remains to be investigated whether circulating CLA^+^ T cells with a skin‐resident transcriptional profile in conditions like psoriasis, AD, and AA are merely bystander cells or drivers of disease pathology. However, prevalence of autoimmune comorbidities and sequential occurrence of skin and distant tissue pathologies in the described inflammatory skin diseases suggest the latter. Collectively, the astonishing heterogeneity and functional plasticity of T_RM_ in inflammatory skin diseases are increasingly understood with the help of high dimensional data obtained by single cell sequencing techniques. Future studies may focus on their migratory potential and re‐seeding properties in disease dissemination and the determination of T‐cell receptor specificities in disease pathogenesis.

### 4.2 Cutaneous T‐cell lymphoma

Mycosis fungoide‐type CTCL is a non‐Hodgkin lymphoma and widely regarded as cancer of mature skin T_RM_.^99^, ^219^ It remains enigmatic whether the disease is rooted in clonal expansion of pre‐existing skin T_RM_,^220^ or skin seeding of circulating neoplastic clones.^221^

Malignant T cells in CTCL share many features with benign skin T_RM_: Dominant CTCL clones are most often CD4^+^ T helper cells expressing the αβTCR, CD69, CD103, and in many cases CCR4.^70^ As with benign skin T_RM_, proliferation is promoted by IL‐15.^220^, ^222^ In advanced CTCL, dominant T‐cell signature shifts from Th2 to Th1 and malignant clones may exhibit loss of TCR or surface receptors CD5, CD7, and CD26.^223^, ^224^ Interestingly, strong inter‐tumor heterogeneity reflects the heterogeneity of skin T_RM_. Unique transcriptomic signatures of the malignant clone are found in each affected patient and sometimes even within different body sites of one individual.^225^, ^226^ Together with a dense benign T‐cell infiltrate, this results in unspecific histopathology, difficult identification of therapeutic targets and diverse clinical presentations resembling inflammatory skin diseases. This constellation of CTCL as “clinical chameleon” leads to prolonged time to treatment and diagnosis remains a challenge. Nonetheless, several treatments have been approved for CTCL: The antibody‐dug conjugate brentuximab vedotin is efficacious in treatment of CD30^+^ CTCL subtypes.^227^ Mogamulizumab conveys antibody‐dependent cell‐mediated cytotoxicity to CCR4‐expressing malignant T cells, but tumor evasion with loss of CCR4 may cause drug resistance.^228^ Allogeneic HSCT is sometimes performed in advanced and leukemic CTCL, but is associated with high relapse rates,^229^ which may be connected to incomplete eradication of host T_RM_ by pre‐transplant myeloablative treatments.^84^, ^98^ Pre‐treatment of CTCL allo‐HSCT recipients with mogamulizumab results in high GVHD incidence.^230^ Other commonly used therapies, including extracorporeal photopheresis and narrow‐band UVB, have not been evaluated in large clinical trials and the mechanism/ effect on malignant T_RM_ populations remains unknown. Overall, the study of CTCL and its treatment options offers interesting insights into T_RM_ biology, resistance and eradication potential.

### 4.3 Therapeutic strategies targeting skin T_RM_

To date, there is no commercially available therapy that specifically targets skin T_RM_ without unwanted side effects on circulating T‐cell subsets or incomplete elimination of sessile populations. Topical and systemic inhibition of Janus kinases (JAK) 1 and 3 transiently abrogates the function of cytotoxic T_RM_ by suppressing their proliferation and cytokine production in vitiligo and AA.^213^, ^231^ On the downside, a significant number of individuals experiences relapse after discontinuation of treatment,^232^ most likely due to persisting pathogenic T_RM_. The IL‐4Rα antibody dupilumab is a highly effective treatment for AD, targeting diverse T helper responses.^233^ However, cessation of therapeutic application frequently results in disease recurrence. Even after long‐term treatment with dupilumab, Bangert et al. show persistence of terminally differentiated CD4^+^ Th2A and CRTAM^+^ cytotoxic CD8^+^ T cells as skin‐resident populations.^234^

In widely used treatments of psoriasis, IL17A inhibition is highly effective in preventing disease but application can rarely be stopped. More recently, anti‐IL‐23 therapies which prevent differentiation of local Th17 cells were approved for psoriasis therapy. Current studies assess whether long‐term treatment could be sufficient to deplete the local T_RM_ pool and prevent recurrence of disease for prolonged periods of time. This has partially been achieved for the treatment of CTCL, where antibody‐dependent cell cytotoxicity against CCR4 depletes malignant CD4^+^ T_RM_ and results in long‐lasting cancer control in some individuals.^235^

Novel treatment approaches focus less on elimination of pathogenic T_RM_ but to re‐balance the skin T‐cell population for example enhancing resident regulatory cell numbers. As T_reg_ were found to enter sites of FDE at resolution of inflammation,^158^ re‐establishment of the local T_reg_ population by desensitization may be a promising approach for the prevention of drug hypersensitivity reactions.^236^ Chimeric antigen receptor T_reg_ represents another recent innovation with the potential to revolutionize treatment of autoimmune disorders,^237^ and preclinical studies have been performed in preventing skin rejection in humanized mice and pathology in vitiligo mouse models.^238^, ^239^ Overall, focused scientific efforts are needed to better understand the role of skin T_RM_ in response to existing treatments and to define new therapies to enhance or dampen their activity in situ.

### 4.4 Conclusion and outlook

The field of skin T_RM_ research has come a long way from initial controversies on the nature of these cells to the appreciation of their heterogeneity and functional plasticity. Initially, T_RM_ were considered a homogeneous group of terminally differentiated CD8^+^ memory T cells expressing CD103, confined to the epidermis.^1^ Today, we understand them as a diverse population of dermal and epidermal memory T cells with stem cell‐like properties and sub‐specializations, “armed and ready” to fight a plethora of different pathogens and malignant cells at the skin barrier site. Local control by T_RM_ in skin cancer has been demonstrated, but their role as targets of immunotherapy is still evolving. Recent reports of T_RM_ plasticity and “inside‐out” responses^63^ indicate promising therapeutic potential of re‐programming and re‐direction of skin T_RM_ in infection and cancer.

While T_RM_ have been well‐characterized as drivers of disease in many inflammatory skin conditions, targeted treatment approaches have rarely been found. The heterogeneity of skin T_RM_ suggests that one size‐fits‐all therapeutic approaches are unlikely to succeed. Future studies on manipulating the local interactions of skin T_RM_ (e.g., with stromal cells) or their metabolic adaptation to the cutaneous environment will provide new insights for therapeutic strategies. Furthermore, we are only beginning to consider the contribution of peri‐ and post‐natal skin‐resident T_reg_ establishment to allergy, autoimmune disorders, and long‐term treatment response in inflammatory disorders. Understanding the homeostatic and pathogenic roles of the heterogeneous T_RM_ pool in human skin will unravel promising therapeutic avenues in the future.

## CONFLICT OF INTEREST STATEMENT

The authors declare no potential commercial or financial conflicts of interest.

## Data Availability

Data sharing not applicable to this article as no datasets were generated or analysed during the current study.
